# Energy Efficiency and Productivity Enhancement of Microbial Electrosynthesis of Acetate

**DOI:** 10.3389/fmicb.2017.00756

**Published:** 2017-05-03

**Authors:** Edward V. LaBelle, Harold D. May

**Affiliations:** Hollings Marine Laboratory, Marine Biomedicine and Environmental Science Center, Department of Microbiology and Immunology, Medical University of South Carolina, CharlestonSC, USA

**Keywords:** microbial electrosynthesis, acetate, hydrogen, chemicals from CO_2_, biocathode, industrial biotechnology

## Abstract

It was hypothesized that a lack of acetogenic biomass (biocatalyst) at the cathode of a microbial electrosynthesis system, due to electron and nutrient limitations, has prevented further improvement in acetate productivity and efficiency. In order to increase the biomass at the cathode and thereby performance, a bioelectrochemical system with this acetogenic community was operated under galvanostatic control and continuous media flow through a reticulated vitreous carbon (RVC) foam cathode. The combination of galvanostatic control and the high surface area cathode reduced the electron limitation and the continuous flow overcame the nutrient limitation while avoiding the accumulation of products and potential inhibitors. These conditions were set with the intention of operating the biocathode through the production of H_2_. Biofilm growth occurred on and within the unmodified RVC foam regardless of vigorous H_2_ generation on the cathode surface. A maximum volumetric rate or space time yield for acetate production of 0.78 g/L_catholyte_/h was achieved with 8 A/L_catholyte_ (83.3 A/m^2^_projected surface area_ of cathode) supplied to the continuous flow/culture bioelectrochemical reactors. The total Coulombic efficiency in H_2_ and acetate ranged from approximately 80–100%, with a maximum of 35% in acetate. The overall energy efficiency ranged from approximately 35–42% with a maximum to acetate of 12%.

## Introduction

The desire to mitigate carbon dioxide emissions (32.1 billion tons CO_2_ emitted globally in 2015) (IEA, 2016) and find sustainable energy sources for increasing global demand have stimulated research in alternative electricity generation and non-fossil fuels. In 2015, 90% of new global electricity generation capacity was renewable (IEA, 2016). With stranded and off peak power, there is a need to balance the energy in the grid and take advantage of every renewable kWh available. One technology that may help address this in the future is microbial electrosynthesis. Utilizing any electricity source, but preferably a sustainable one, different anaerobic microbes have produced several chemicals of interest by using CO_2_ as the sole carbon source when operating at the cathode of an electrochemical cell (Nevin et al., 2010; Logan and Rabaey, 2012; Marshall et al., 2012, 2013; Lovley and Nevin, 2013; LaBelle et al., 2014; Blanchet et al., 2015; Jourdin et al., 2015; Schröder et al., 2015; Ammam et al., 2016; Deutzmann and Spormann, 2016).

Ultimately, products from microbial electrosynthesis would include high value chemicals and liquid fuels. However, simply producing a single C-C bond such as in acetate would be worthwhile and much of the work done thus far has focused on electroacetogenesis (Conrado et al., 2013; May et al., 2016). Closely related to this is the bioconversion of syngas (H_2_, CO, and CO_2_) to fuels and chemicals (Daniell et al., 2012, 2016; Hu et al., 2013, 2016), and within this area of research is the study of H_2_:CO_2_ transformation to acetate by acetogenic bacteria. Acetate itself is of commercial importance and it can be transformed further into other products (Andersen et al., 2014; Hu et al., 2016; Pal and Nayak, 2016). For these reasons there has been a significant effort recently to improve the rates and efficiency of acetogenesis with H_2_:CO_2_ or bioelectrochemically.

Previous work in gas fermentations has illuminated reaction conditions that can limit or enhance the rates of acetogenesis. Demler and Weuster-Botz (2011) achieved a volumetric productivity or space time yield (STY) of 0.31 g/L/h with *Acetobacterium woodii* in a batch stirred tank reactor pressurized with H_2_:CO_2_. Later Kantzow et al. (2015) achieved an impressive STY of 6.16 g/L/h in continuous flow stirred tank reactors (CSTR). The investigators demonstrated that increasing the dilution rate and increasing biomass retention led to higher acetate productivity. Hu et al. (2013) showed that in bubble columns, electron transfer to *Moorella thermoacetica* was limited by the mass transfer of CO into solution at rates below 30 mM/h, which is a volumetric current density equivalent to 1.6 A/L. Hu et al. (2016), an improved design led to a STY of 1.1 g/L/h of acetate from H_2_:CO_2_ by *M. thermoacetica* first grown with CO. The investigators integrated the process with *Yarrowia lipolytica* to produce biodiesel precursors, thereby demonstrating the feasibility of linking syngas fermentation with an acetate-consuming biofuel-producing process. Overall, these investigations indicate that fuel and chemical production could be driven by H_2_ gas fermentations at industrially relevant rates. Could this be accomplished with microbial electrosynthesis?

The objective of this study was to improve the productivity and efficiency of microbial electrosynthesis through reactor design and operation (galvanostatic mode, H_2_ mediation, continuous flow). Data from previous studies with an acetogenic community used in bioelectrochemical systems with graphite granule cathodes (Marshall et al., 2012, 2013; LaBelle et al., 2014) generally indicated that the early reactors were of an inefficient design and that more biomass at the cathode was likely needed to improve performance. Patil et al. (2015) discussed and demonstrated the utility of delivering electrons in a MES with galvanostatic operation, and this also resulted in faster startup times and improved scalability. Instead of waiting for higher rates of H_2_ evolution from the electrode as in previous MES studies operated potentiostatically, here it was hypothesized that using a high volumetric current density surpassing the 1.6 A/L elucidated from Hu et al. (2013) would result in shorter startup times, faster biomass growth rate, and faster acetate production. Biomass growth would also benefit from a continuous flow of nutrients, and a nitrogen limitation was indicated by the upregulation of nitrogen fixing enzymes in batch-fed bioelectrochemical reactors (Marshall et al., 2016). Continuous flow of media will also alleviate product inhibition (Demler and Weuster-Botz, 2011; Kantzow et al., 2015), decrease the reactor footprint, and facilitate pH control; this acetogenic community has an acetate and H_2_ product ratio that is affected by pH in potentiostatic systems (LaBelle et al., 2014). Thus, galvanostatic control and continuous flow were applied to a reactor designed for better energy efficiency and STY, which are also important to decrease capital and operating costs (Krieg et al., 2014; Papoutsakis, 2015). The goal was to develop a system that could be used to reach g/L/h productivity while maintaining a high energy efficiency. This was done with high current density normalized to surface area (projected and geometric). However, since the acetate product is soluble, the volumetric current density is important when considering the reactor productivity as well as product titer needed to devise an efficient extraction scheme (Krieg et al., 2014; Gildemyn et al., 2015; Papoutsakis, 2015; Patil et al., 2015). The approach resulted in a considerable enhancement of productivity and efficiency.

## Materials and Methods

### Bioelectrochemical Reactor

The modular and scalable reactors were designed and constructed as depicted in Supplementary Figures S1A,B. The 45 pores per inch (17.7 pores per cm) reticulated vitreous carbon (RVC) foam cathode (KR Reynolds Company) was 0.6 cm × 6 cm × 8 cm (surface area to volume ratio of 26.2 cm^2^/cm^3^ per manufacturer). It was pretreated in 2N nitric acid and rinsed thoroughly with MilliQ water. It was then attached to a 6 × 6 mesh, 0.89 mm wire diameter 316L stainless steel mesh (6 cm × 8 cm) current collector that was coated with a conductive graphite and carbon black acrylic glue (Ted Pella Inc. #16050) thinned with 1:1 (v/v) acetone. Two applications were coated onto the mesh before the same glue was used to attach the RVC foam. The anode was a mixed metal oxide (IrO_2_/Ta_2_O_5_) catalyzed titanium anode (MMO; Magneto).

Custom machined polypropylene spacers were used with customized Viton gaskets to sandwich a cation exchange membrane (CMI-7000, Membranes International Inc.) between the electrodes with a 316L stainless steel endplate and a poly(methyl methacrylate; PMMA) cathode viewing plate held together by stainless steel nuts, bolts and washers. The polypropylene spacers were 0.95 cm × 10 cm × 10 cm, with a square opening of 8 cm × 8 cm. The steel plate was 0.95 cm × 15.2 cm × 15.2 cm. The PMMA cathode viewing plate was 1.25 cm × 15.2 cm × 15.2 cm. The membrane and exposed anode surface area were each 64 cm^2^. The projected cathode surface area was 48 cm^2^. Polypropylene Luer fittings (McMaster Carr) were used as ports to connect tubing. Reference electrodes were constructed using a AgCl coated silver wire immersed in 3M KCl saturated with AgCl in a glass capillary tube with a Vycor frit attached via heat shrink tubing. A Luggin capillary was integrated in the reactor using 1 M KCl as supporting electrolyte, and its Vycor frit was ∼2 mm from the cathode.

The anolyte was 75 mL of 50 mM sodium sulfate acidified with sulfuric acid to pH = 2. The catholyte was 50 mL of a phosphate-based medium initially prepared at pH 7 under 100% N_2_ as described in LaBelle et al. (2014). It contained salts, vitamins and trace metals and minerals. 50 mM NaCl was used instead of sodium bromoethanesulfonate. No yeast extract, sulfide or cysteine was used.

### Reactor Operation

An acetogenic microbiome previously enriched on graphite granule cathodes at potentials from -590 to -800 mV vs. SHE was used to inoculate the bioreactors (LaBelle et al., 2014). The cells were drawn from the cathode compartment of an active reactor and were concentrated using tangential flow filtration using a 0.2 μm polyethersulfone filter (Sartorius), spun at 5000 (RCF) for 10 min and re-suspended in fresh medium before transfer to the RVC cathode reactors. The culture, inoculum and reactor, were treated as an open, non-aseptic system.

The reactor was operated at 25°C with constant current supplied from a VMP3 Potentiostat (BioLogic) set in galvanostatic mode and the voltage monitored with EC Lab software. A galvanostatic operation of 8 A/L_catholyte_ was chosen to overcome electron limitation to a biofilm immobilized on the cathode and promote fast colonization. Humidified 100% CO_2_ was passed through the catholyte headspace using Norprene tubing at an initially set rate of 25 mL/min. Filter sterilized medium was flowed into the base of the cathode compartment and exited just above the top of the RVC cathode at a rate of 250 mL/day (a dilution rate of 5 day^-1^) using a peristaltic pump and PharMed BPT tubing. Deionized water was added to ports made from syringe housing on the anolyte compartment daily to compensate for water oxidation and evaporation.

### Sampling and Analysis

The gas flow rate was monitored with an Agilent gas flow meter when sampling headspace for gas chromatographic analysis, and fatty acids were analyzed via HPLC (LaBelle et al., 2014). The pH of samples drawn from the cathode chamber was checked with a pH meter (Mettler Toledo). Optical density of the planktonic cells (OD_600nm_) was measured at 600 nm using a Genesys UV/Vis spectrophotometer.

Space time yield was calculated as the STY = concentration times flow rate divided by catholyte volume (Krieg et al., 2014). The Coulombic efficiencies were calculated from the partial current densities of each product divided by the total applied current. Energy efficiencies were obtained by using the higher heating value (HHV) of product multiplied by the STY and then divided by the applied electrical energy to the reactor per time (Krieg et al., 2014).

## Results and Discussion

### Biomass Growth

**FIGURE 1:**
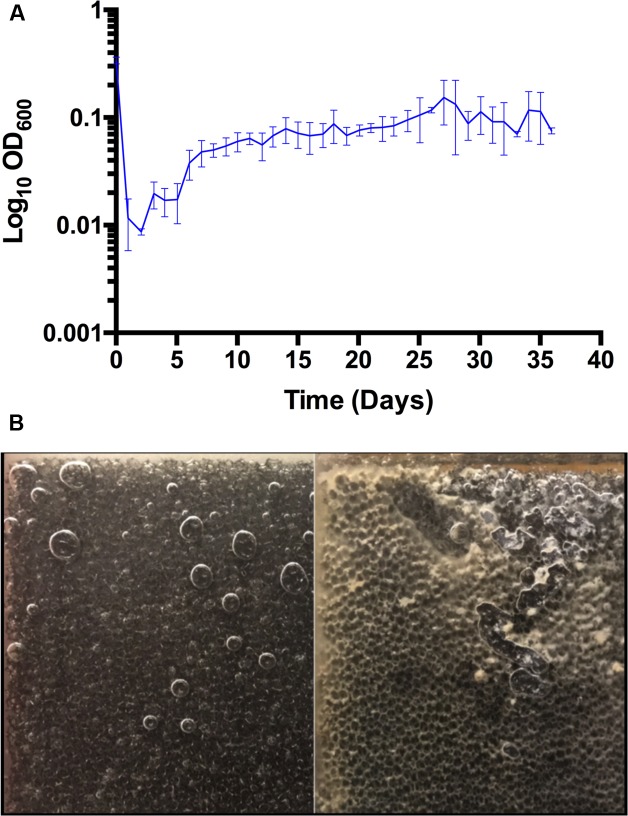
**(A)** Optical density (600 nm) of planktonic cells in triplicate bioelectrochemical reactors (SD, *n* = 3). **(B)** Biofilm growth on RVC cathode. Photographs of uninoculated (left) and inoculated (right) 45 ppi RVC cathodes at 36 days.

The initial inoculum resulted in an optical density (OD_600nm_) of 0.34 ± 0.01 (*n* = 3) within the cathode chamber of three inoculated reactors (**Figure 1A**). The continuous flow of media through the reactors, and perhaps adsorption of biomass to the electrode surface, drove the OD_600nm_ down by more than an order of magnitude. Shortly thereafter the OD_600nm_ began to steadily increase and eventually remained near or above 0.1 within the reactors, indicating a constant production of bacterial cells including those that remained planktonic and were washed away with the effluent. A coating of the unmodified cathode surface with yellow and off-white material became apparent within a week of inoculation and grew heavier until the end of the experiment (**Figure 1B**). Microscopic examination of the material from the electrode surface and from within the interstitial space revealed that it was densely populated with bacterial cells similar to *Acetobacterium* in morphology, the genus that dominated the inoculum (LaBelle et al., 2014). Although the biomass on the electrodes was not quantified, visually it was apparent that the colonization of the electrode continued until the experiment was terminated.

While graphite granule cathodes were effective at enriching the community (Marshall et al., 2012, 2013; LaBelle et al., 2014), and are a relatively inexpensive and high surface area electrode for electroactive biofilms, their drawbacks include gas hold up, high resistance, high granular void volume, and restriction of liquid flow for continuous operation, thus warranting a different electrode geometry. RVC was chosen because of its high surface area to volume ratio, low solid void volume due to the glassy carbon (97% air-liquid void volume), and this electrode material performs well for flow through systems (Krieg et al., 2014). Unmodified RVC has been reported to be a poor surface for bioelectrochemical systems (Flexer et al., 2013), and it did not support the electrosynthesis of any acetate with another microbial community (Jourdin et al., 2014) unless its glassy carbon surface was coated with carbon nanotubes (Jourdin et al., 2015). For the latter case a current density of 0.055 A/L (102 A/m^2^_projected surface area_) and an acetate rate of 0.015 g/L/h and electron recovery of 100% were achieved under poised potential conditions in a batch-fed reactor. In contrast, the microbial community used under the conditions described here (8 A/L, 83 A/m^2^_projected surface area_) readily colonized the unmodified RVC and generated acetate (see below) with this material serving as a biocathode. Microbubbles vigorously effervesced throughout and larger bubbles coalesced on the cathodes during the start-up. One may expect this to limit or even prevent the formation of a biofilm on the surface and within the honeycomb of the cathode (Blanchet et al., 2015; Bergel, 2016). However, biofilm became clearly visible on the cathodes following inoculation (confirmed by optical microscopy) while no visible changes were observed for more than a month with uninoculated RVC maintained under the same conditions. It is likely that biological and non-biological material was deposited on the cathode, but this was only visible when the cathodes were inoculated and the key to improved productivity was probably due to the increase in biomass on and within the RVC. Furthermore, there were no indications at the end of the experiment that the biomass/biofilm could not be increased further, which may additionally improve productivity and efficiency. How this biofilm forms and how it influences hydrogen formation and transfer, acetogenesis, and overall performance will require further inquiry.

### Productivity

**FIGURE 2:**
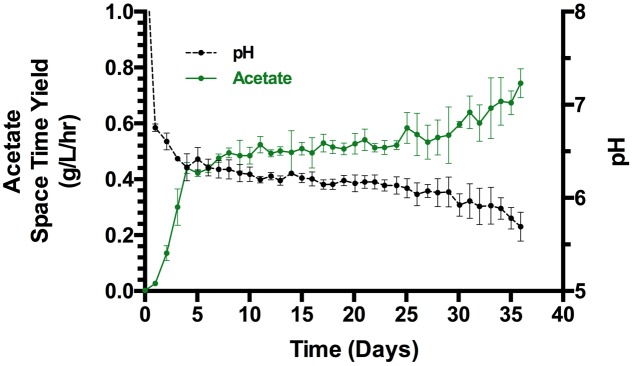
**Space time yield of acetate and pH of triplicate bioelectrochemical reactors (SD, *n* = 3)**.

The STY of acetate was monitored in the three inoculated continuous flow/constant current reactors (**Figure 2**). Within the first 4 days of operation, the STY rapidly increased to >0.4 g/L_catholyte_/h. From this point forward the rate steadily increased and was accelerating at the end of the experiment (day 36) when the STY reached a maximum of 0.78 g/L_catholyte_/h, 0.74 ± 0.05 g/L_catholyte_/h in replicates (*n* = 3). The maximum acetate produced per electrode surface area was 8.2 g/m^2^_projected_/h. Hydrogen was co-produced at 0.2 g/L_catholyte_/h at the end of the experiment. Other products included formate, propionate, and butyrate produced at <0.02 g/L_catholyte_/h (Supplementary Figure S2). No methane was detected and biomass growth and acetate production were sustained without the addition of methanogenic inhibitors, yeast extract, or chemical reducing agents.

Shortly after inoculation, the pH of the biotic reactors rose abruptly to 8.88 ± 0.53 due to the production of base at the cathode of the electrochemical cell. However, the continuous supply of CO_2_ and medium flow returned the pH to 6.75 ± 0.03 within 24 h. The pH decreased as the microbial production of acetic acid increased and the change in pH was more rapid at the end of the experiment. A single uninoculated reactor was maintained under the same conditions as the three inoculated reactors (Supplementary Figure S3). After an initial upward spike in pH during the 1st day, the pH stabilized at 6.67 ± 0.03 for the remainder of the experiment.

### Efficiency

**FIGURE 3:**
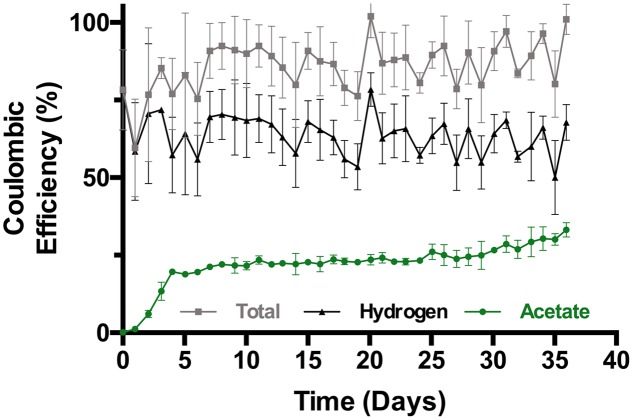
**Coulombic efficiency of triplicate bioelectrochemical reactors (SD, *n* = 3)**.

Following the initial start-up, the overall Coulombic efficiency (CE) ranged from approximately 75–85% with about 20% in acetate (**Figure 3**). From then to the end of the experiment the overall CE ranged between approximately 80 and 100% with most of the variability in the measurement of H_2_ (the CE for the single uninoculated control reactor was 88%). The electron recovery in acetate steadily increased to 33.2% ± 2.3% (*n* = 3) at day 36 with 35% in one reactor.

**FIGURE 4:**
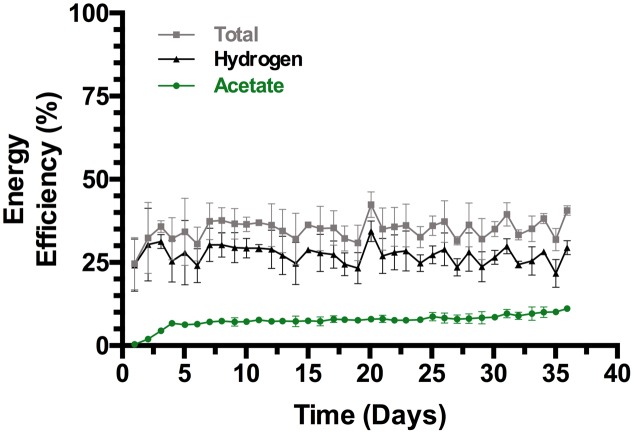
**Energy efficiency of triplicate bioelectrochemical reactors (SD, *n* = 3)**.

The trend in energy efficiency (**Figure 4**) generally followed that of the CE (**Figure 3**), and both efficiencies paralleled the increase in the STY of acetate (**Figure 2**). Following the initial increase in productivity during the first 4 days of incubation the energy efficiency fluctuated between 30 and 35% with approximately 7% invested in acetate. From then to the end of the experiment, the overall energy efficiency varied between 35 and 42% while the energetic efficiency in acetate steadily increased to 11.2% ± 0.8% (*n* = 3) with a 12.1% maximum in one reactor at the end of the experiment. Both the CE and energy transferred to acetate were increasing at the end of the experiment.

Voltage ranged from 3.2 to 3.6 over time with all three reactors, and potential at the cathodes was -1.1 to -1.3 V vs. SHE (Standard Hydrogen Electrode) with no clear trend up or down. The power consumption of the reactors is depicted in Supplementary Figure S4. Better electrode spacing and a more efficient anode may improve the efficiency of the reactors. What would likely most improve the efficiency to acetate for these bioelectrochemical reactors is to avoid the loss of H_2_; this may be related to retaining more biomass in the reactor. Adding pH control and additional cell retention to the system may be the solution. The RVC is enabling some cell retention in the reactor in addition to efficient electron delivery, but the contribution from cells on the electrode vs. within the honeycomb of the electrode or suspended in the medium is unknown, and there is a significant fraction of planktonic biomass that is presently lost with the continuous flow (∼0.1 OD_600nm_). Hence further cell retention in the cathode chamber may additionally enhance productivity and efficiency and the results warrant a more detailed examination of the biofilm formation on the cathodes in relation to productivity and efficiency. For example, attaining a 100% CE to acetate under the current presently applied could result in a STY of 2.2 g/L/h and an energy efficiency >30% to acetate.

### Microbial Electrosynthesis vs. Gas Fermentations

When comparing the supply of electrons (H_2_) supplied to gas-liquid contacting (GLC) bioreactors (Demler and Weuster-Botz, 2011; Hu et al., 2013, 2016; Kantzow et al., 2015), it became apparent that the electrons supplied to the bioelectrochemical reactors in the past (Marshall et al., 2012, 2013; LaBelle et al., 2014) were limited and this precluded any chance of high productivity and likely blunted the growth of biomass; hence the remedy described above. Abiotic and biotic activity may functionalize the surface of the cathode, thereby facilitating the production of H_2_ or formate for the eventual generation of acetate and other products (LaBelle et al., 2014; Yates et al., 2014; Deutzmann et al., 2015; Marshall et al., 2016; May et al., 2016), and the results presented here were dependent upon the addition of the microbial community, but the electrochemical bioreactors were intentionally operated through H_2_ with the goal of improving productivity and efficiency. Therefore, it is reasonable to compare microbial electrosynthesis with H_2_ gas fermentations (Blanchet et al., 2015; Tremblay et al., 2016).

Microbial electrosynthesis may be indirectly approached by the coupling of a H_2_ producing electrolyzer with a GLC bioreactor such as a CSTR or a continuous flow bubble column where H_2_ is delivered to a fuel or chemical producing microbe (e.g., an acetogen). Alternatively, during direct MES the microbes are incubated with the cathode of an electrochemical cell where they capture H_2_ or possibly electrons directly from an electrode (Butler and Lovley, 2016; Tremblay et al., 2016). Productivity and efficiency data from these two approaches are presented in **Table 1**. A 70% energy efficiency for an electrolyzer (Körner et al., 2015), based on a HHV of H_2_, was assumed for calculating the efficiency of an indirect MES with two reactors. Following growth with CO in a continuous flow bubble column with cell retention, the thermophile *M. thermoacetica* produced acetate at 1.1 g/L/h when supplied with H_2_:CO_2_, but there was a trade-off with efficiency when examining it as part of an indirect MES. The *M. thermoacetica* system was designed for use with hot syngas and the high temperature (60°) lowered the solubility of the H_2_ (Hu et al., 2013, 2016), which required high sparging rates to overcome mass transfer and reduced efficiency.

**Table 1:** Indirect vs. direct microbial electrosynthesis of acetate.

Organism	Reactors		Temp. (C)	Dilution rate (d^-^^1^)	Yeast extract (g/L)	HFM^#^	pH stat^∗^	Acetate (g/L/h)	Acetate electron recovery	Acetate energy efficiency	Reference
*Moorella thermoacetica*	70% EE electrolyzer	Bubble column	60°	2.16	10	Yes	Yes	1.1	3.7%	2.0%	Hu et al., 2016
*Acetobacterium woodii*	70% EE electrolyzer	CSTR	30°	0.84	4	No	Yes	0.76	10.3%	4.6%^a^	Kantzow et al., 2015
*Acetobacterium woodii*	70% EE electrolyzer	CSTR	30°	8.4	4	Yes	Yes	6.16	55.9%	26.4%^a^	Kantzow et al., 2015
*Acetobacterium sp.* (mix)	Bioelectrochemical constant current^b^		25°	5	0	No	No	0.78	35%	12.1%	This study
Clostridiales (mix)	Bioelectrochemical constant current^c^		21°	Batch	0	No	No	0.024	61%	21%	Gildemyn et al., 2015
*Sporomusa ovata*	Bioelectrochemical constant voltage^d^		25°	0.0144	0	No	No	0.0009	105%	50%	Giddings et al., 2015


*Acetate electron recovery for indirect MES was based on electrons in acetate produced divided by the total electrons sparged as H_*2*_ through the reactor from the electrolyzer. Acetate energy efficiency was calculated using the HHV of acetate produced divided by the energy to power an electrolyzer that has a 70% energy efficiency (EE) based on the HHV of H_*2*_ produced. ^a^Total energy applied included energy required for stirring the CSTR. ^b^Applied Current: 8 A/L_*catholyte*_ (83.3 A/m^*2*^_*projected*_). ^c^Applied Current: 0.143 A/L_*catholyte*_ (5 A/m^2^_*projected*_). ^d^Applied Voltage: 1.9 V. Hollow fiber membranes are used for full cell retention. ^*#*^Hollow fiber membrane. ^∗^pH auxostat system.*

When assessed as an indirect MES, a CSTR with *A. woodii* (Kantzow et al., 2015) produced acetate at nearly the same rate (0.76 g/L/h) but with substantially higher efficiency than *M. thermoacetica* in a bubble column. The highest acetate productivity (6.16 g/L/h) in combination with a high CE (55.9%) and energy efficiency (26.4%, estimated as an indirect MES including stirring energy) was achieved with *A. woodii* incubated in a CSTR with cell retention and a high rate of dilution (Kantzow et al., 2015). This energy efficiency does not match the 39–50% reported for a membraneless direct MES reactor operated in batch under constant applied voltage (Giddings et al., 2015), but due to its productivity the CSTR coupled with an electrolyzer is for now a more feasible approach. The energy efficiency of the direct MES described in the present study does exceed many of these other systems while maintaining high productivity, but its performance does not yet match that of the CSTR run with cell retention and a high dilution rate. However, as noted above the productivity and efficiency of the direct MES may be improved with more pH control and cell retention.

The results described here indicate the productivity and efficiency of a direct MES operating through H_2_ in a single reactor is approaching that of an indirect MES using two reactors. Furthermore, rates of acetogenesis from H_2_:CO_2_ within GLC bioreactors are now on par with the productivity of ethanol fermentation and direct MES is approaching it. Starch (corn) ethanol is commercially produced at 1.25 to 3.75 g/L_reactor volume_/h (Graves et al., 2006; Richter et al., 2013), but this rate of production ignores the time, arable land, and energy needed to grow, harvest, transport, and pretreat the substrate prior to fermentation (Fast and Papoutsakis, 2012; Conrado et al., 2013). Ethanol and acetate do not possess the same energy or commercial value and absolute comparisons of their production cannot be made. But the aforementioned comments are insightful since bioethanol from starch is the largest biorefinery process scaled to date, and if electrosynthesis of acetate or any other chemical is to ever be viable then productivity must be within the range of bioethanol production.

### Cost, Titer, and Extraction

At the efficiency and rates described here for electroacetogenesis, 33.6 kWh are needed to produce 1 kg of acetate with a direct MES and 15.4 kWh with an indirect MES using a CSTR. At $0.02 per kWh (national average levelized price of wind power purchase agreements) (Wiser and Bolinger, 2016), the electricity cost for 1 kg of acetate would range from $0.31 to $0.67; as with any system, the need for extraction and purification would undoubtedly add to this cost (Gildemyn et al., 2015; Papoutsakis, 2015; Xu et al., 2015). The global price of acetic acid has ranged from $0.35 to $0.95 per kg between 2010 and 2015 (Wakatsuki, 2015), while U.S. domestic acetic acid spot pricing was at $0.49 per kg and U.S. spot export pricing was at $0.60–$0.63 per kg in September of 2016 (Raizada, 2016). Prices from $0.90 to $1.60 per kg were also reported in a variety of countries during 2015 and 2016 (Nayak and Pal, 2015; Nayak et al., 2015; Pal and Nayak, 2016). While these quotes indicate a range of possible commercial values for acetate, they also show that further improvements in electrosynthesis performance remain desirable. Selling the excess hydrogen and oxygen simultaneously produced could attain additional revenue, and increases in efficiency will help. For example, achieving 100% CE under the same conditions in this work would lower the energy requirement to 12 kWh per kg, with $0.24 in electricity costs per kg acetate produced. However, titer is another factor that affects purification costs. The titer reached 3.6 g/L in the present experiment and 10.5 g/L in past batch experiments with the same microbial community (Marshall et al., 2013). Optimizing the dilution rate in relation to nutrient supply, pH control, and cell retention will increase the titer in the continuous flow/constant current reactors, but significantly higher titer still needs to be achieved.

A promising approach to increase titer has been demonstrated by Gildemyn et al. (2015), who reported on a galvanostatic microbial electrosynthesis system in batch mode that produced acetate at 0.024 g/L/h, and simultaneously extracted the acetate across an anion exchange membrane to concentrate the product in an integrated extraction compartment of equal volume to the catholyte. This method obviates the need for an external pH control system, and the 13.5 g/L titer achieved with this method is the highest reported for a direct MES. The energy efficiency to acetate, including the extraction, reached an impressive 21%.

Another possible solution is to supply the acetate to a second bioreactor that produces higher value products that may be produced at a higher titer and are more readily extracted, such as demonstrated by Hu et al. (2016) for the production of biodiesel precursors. Chemocatalytic upgrading has also been used to produce ethyl acetate by combining biphasic esterification with an integrated electrolytic membrane extraction of acetate from fermentation of thin stillage biomass (Andersen et al., 2014). This process was also adopted to utilize acetate made from electricity and CO_2_ to produce the ethyl acetate (Andersen et al., 2016).

### Additional Considerations

Yeast extract has been required for the growth of acetogens in some studies (Kantzow et al., 2015; Hu et al., 2016), and at the concentrations and dilution rates used would be cost prohibitive (Lawford and Rousseau, 1997; Lau et al., 2012). A metagenomic analysis indicated that the microbial community used here possesses the full complement of genes required for the synthesis of all amino acids and vitamins needed for growth (Marshall et al., 2016), and yeast extract has never been used with this community.

The experiments reported here were done with a phosphate buffer, and to continuously supply that would also be cost prohibitive at the dilution rates in this study. Past batch-fed experiments were done with a bicarbonate buffer in place of the phosphate (LaBelle et al., 2014), which would be far more cost effective (est. 80% media cost reduction).

Both hydrogenotrophic and acetoclastic methanogens would divert electrons away from acetate synthesis. Inhibition of methanogenesis can be costly, but no inhibitor was needed for this experiment or for preparation of the inoculum. Operating a direct MES reactor through H_2_ under galvanostatic control may offer a competitive advantage for the acetogens versus methanogens in that high H_2_ partial pressure favors acetogenesis. Furthermore, allowing the pH to drop below 6 may also favor the acetogens since lower pH can also inhibit methanogenesis (Ni et al., 2010; Zhang et al., 2013; Spirito et al., 2014). Methanogens initially present in the community enriched for this biocathode were eliminated by repeated exposure to high H_2_, low pH, and the addition of the inhibitor sodium bromoethanesulfonate (NaBES) (Marshall et al., 2012, 2013; LaBelle et al., 2014). The use of NaBES with this microbial community was ended in 2014, it was not used at any time in this study, and the inoculum used was transferred more than three times without NaBES before use in this study. The community used in this study has always been maintained in an open system (non-aseptic) in a laboratory with methanogenic cultures and sediments under investigation nearby.

The scalability and economics of MESs has been addressed in several studies and reviews (Foley et al., 2010; Desloover et al., 2012; Logan and Rabaey, 2012; Conrado et al., 2013; Brown et al., 2014; Harnisch et al., 2014; ElMekawy et al., 2016; Zhang and Angelidaki, 2016). Additionally, current legislation concerning whether the CO_2_ is obtained from renewable or waste gasses can vary geographically (Daniell et al., 2016; Dürre, 2016). This can affect whether the product can qualify for credits and incentives, and thus be sold competitively. However, a life cycle analysis has shown that ethanol produced from CO_2_ recycled from a fossil fuel waste stream can still attain a net carbon emission reduction (Daniell et al., 2016; Handler et al., 2016). Further improvements in performance are still desired and product selection should go beyond acetate, but with productivity and efficiency for a direct single reactor MES approaching the best reported for an indirect MES with two reactors, successful scaling and improved performance of microbial electrosynthesis is now more feasible. Building and operating one reactor versus two may reduce capital and operating costs, and the performance of the reactor systems for acetogenesis compared in **Table 1** suggest such a consolidation warrants further consideration. What is needed is a systematic laboratory comparison of direct and indirect MES systems. Such data will help develop practical process systems and better inform life cycle assessments and technoeconomic analyses to contribute to proper implementation of MES.

## Conclusion

This work contributes practical operating conditions for microbially producing acetic acid from electricity and carbon dioxide, with hydrogen as co-product, bringing the technology closer to real world implementation. Additionally new benchmarks in STY and efficiency are presented. Acetate was produced at a maximum STY of 0.78 g/L/h, and in replicate 0.74 ± 0.05 g/L/h (*n* = 3). The growth of this technology and its promise in complementation to other established and emerging technologies indicate the viability of microbial electrosynthesis to aid in carbon and energy management.

## Author Contributions

EL and HM designed and planned the research, EL conducted the experiments, and EL and HM analyzed the data and wrote the manuscript.

## Conflict of Interest Statement

Patents pending in relation to this work: 1. Harold D. May, Marshall CW, and Edward V. LaBelle. Microbial Electrosynthetic Cells. PCT/US2013/060131. International filing date: 17 September 2013. 2. 1. Harold D. May and Edward V. LaBelle. Provisional Application for United States Letters Patent for Bioelectrosynthesis of Organic Compounds. November 3, 2016.
