# Supplementary material for: Energy Efficiency and Productivity Enhancement of Microbial Electrosynthesis of Acetate
Source: Front Microbiol. 2017 May 3;8:756. doi: 10.3389/fmicb.2017.00756 (PMC5413574; doi:10.3389/fmicb.2017.00756)
Supplement: Supplementary file 1 [file Data_Sheet_1.PDF]

Supplementary Material

Energy Efficiency and Productivity Enhancement of Microbial Electrosynthesis of Acetate

Edward V. LaBelle and Harold D. May

*** Correspondence:** Corresponding Author: mayh@musc.edu

A

B

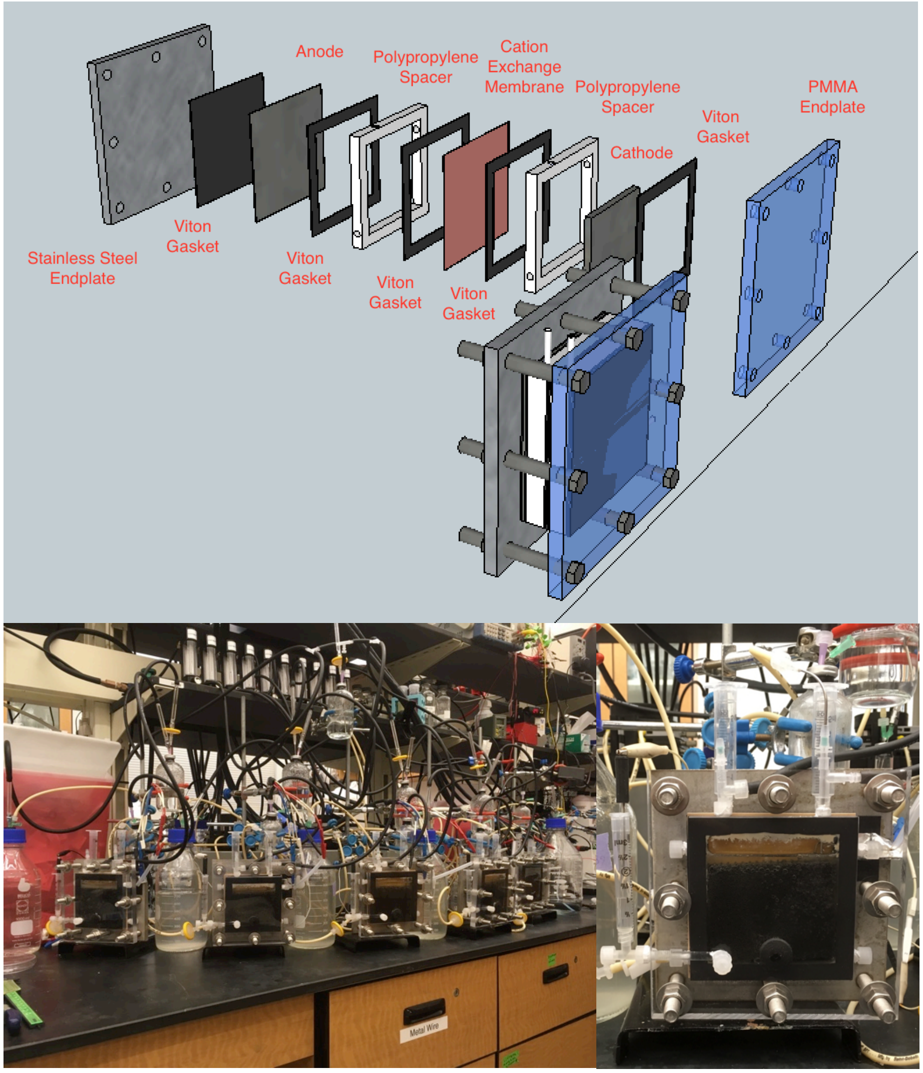

Figure S1. (A) Three-dimensional rendering of components and assembly of the bioelectrochemical reactor. (B) Constructed continuous flow bioelectrochemical reactors. See Materials and Methods Section for dimensions and details.

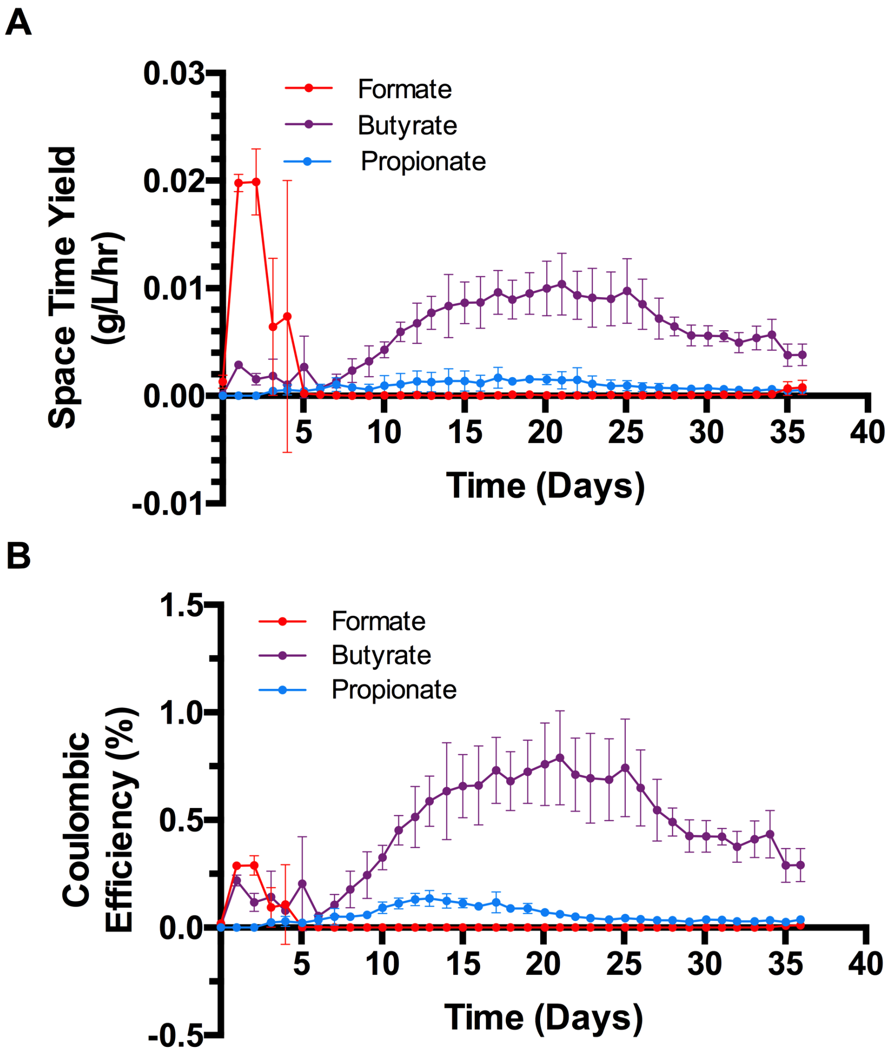

Figure S2. (A) Space time yield and (B) Coulombic efficiency of secondary products produced in triplicate bioelectrochemical reactors (SD, n=3).

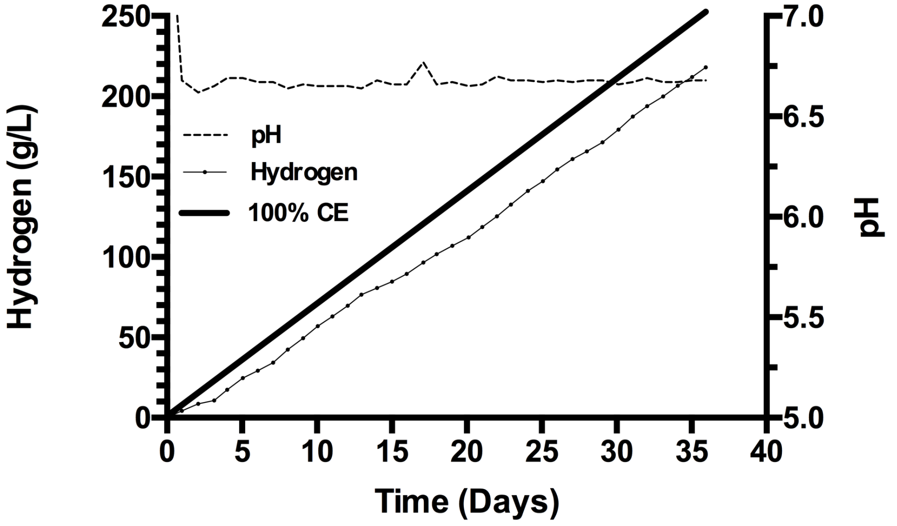

Figure S3. Hydrogen production of an uninoculated abiotic reactor operated under the same conditions as biotic reactors.

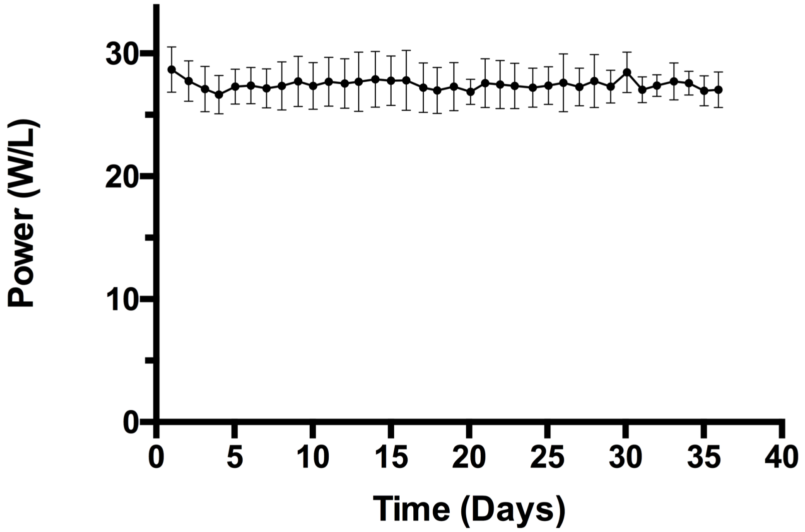

Figure S4. Power consumption of triplicate bioelectrochemical reactors (SD, n=3).
